# Cellulosic Ethanol Production by Recombinant Cellulolytic Bacteria Harbouring *pdc* and *adh* II Genes of *Zymomonas mobilis*

**DOI:** 10.1155/2012/817549

**Published:** 2012-07-20

**Authors:** P. Sobana Piriya, P. Thirumalai Vasan, V. S. Padma, U. Vidhyadevi, K. Archana, S. John Vennison

**Affiliations:** Department of Biotechnology, Anna University of Technology, Tamil Nadu, Tiruchirappalli 620024, India

## Abstract

The ethanol fermenting genes such as pyruvate decarboxylase (*pdc*) and alcohol dehydrogenase II (*adh* II) were cloned from *Zymomonas mobilis* and transformed into three different cellulolytic bacteria, namely *Enterobacter cloacae* JV, *Proteus mirabilis* JV and *Erwinia chrysanthemi* and their cellulosic ethanol production capability was studied. Recombinant *E. cloacae* JV was found to produce 4.5% and 3.5% (v/v) ethanol, respectively, when CMC and 4% NaOH pretreated bagasse were used as substrates, whereas recombinant *P. mirabilis* and *E. chrysanthemi* with the same substrates could only produce 4%, 3.5%, 1%, and 1.5 % of ethanol, respectively. The recombinant *E. cloacae* strain produced twofold higher percentage of ethanol than the wild type. The recombinant *E. cloacae * strain could be improved further by increasing its ethanol tolerance capability through media optimization and also by combining multigene cellulase expression for enhancing ethanol production from various types of lignocellulosic biomass so that it can be used for industrial level ethanol production.

## 1. Introduction 

The conversion of plant cellulose biomass to fuel ethanol by microbial fermentation is the priority area of research, and the use of industrially suited microorganisms for the cost-effective biofuel production is the major technical challenge. Cellulosic ethanol would reduce our petroleum dependency, as ethanol is produced from the inexpensive and plentiful feed stocks. Efficient conversion of biomass to ethanol requires development of microorganisms capable of fermenting a wide range of carbohydrates and tolerating high concentrations of ethanol [1]. Metabolic engineering of microorganisms to utilize cellulose will be vital for improving the prospects of significant cellulosic ethanol production. Several Gram-negative bacteria such as *Escherichia coli*,* Klebsiella oxytoca,* and* Zymomonas mobilis* have been engineered for ethanol production [2–5]. 

Enteric bacteria normally produce less ethanol, because of their poor efficiency in converting pyruvate to ethanol. A suitable ethanologenic and cellulose-producing bacteria could be developed by transferring genes that encode the ethanol-fermenting enzymes [6]. * Z. mobilis* is one of the best ethanol producers which produces ethanol in the Entner-Doudoroff (ED) pathway, that is, homoethanol fermentation pathway with the help of two essential enzymes such as pyruvate decarboxylase (PDC) and alcohol dehydrogenase (ADH) encoded by* pdc* and* adh* II genes, respectively. PDC catalyzes the nonoxidative decarboxylation of pyruvate to produce acetaldehyde and carbon dioxide, whereas ADH catalyzes the reduction of acetaldehyde to ethanol during fermentation [7–9]. These two enzymes (both PDC and ADH) are sufficient to convert intracellular pool of pyruvate and NADH to ethanol [10]. 

The transfer of ethanol-fermenting genes (*pdc* and* adh*) from* Z. mobilis* to cellulolytic bacteria could definitely improve their ethanol productivity by converting pyruvate completely to ethanol. The research on the construction of recombinant ethanol fermenting bacteria by expressing both the* pdc* and* adh* II genes was originally done by Ingram et al. [9] in* E. coli* to change the ethanol production ability by fermenting all sugars in the biomass. Similarly, recombinant* Erwinia* sp. [11] and* Klebsiella oxytoca* M5A1 [2] were developed to improve the ethanol production from xylose and glucose. Recombinant Gram-negative* E. coli* KO11 [1] and Gram-positive* Clostridium cellulolyticum* [12] were constructed to produce ethanol from acid hydrolysates of hemicellulose and lignocellulosic biomass, respectively. Though these reports did explain the cloning of* pdc* and* adh* genes, but the subsequent usage of the cloned genes for ethanol production was not explained clearly. The formation of additional byproducts during fermentation and tolerance to the produced ethanol are the major limitations observed in these studies. The bioethanol production from cellulosic biomass in cellulolytic microorganisms can be improved by introducing ethanol-fermenting genes under the control of an appropriate promoter [9]. 

In the present study, the ethanol fermenting genes such as* pdc* and* adh* II were cloned from *Z. mobilis* and introduced into three facultative anaerobic, Gram-negative cellulolytic bacteria. The cellulosic ethanol production capability of these recombinant strains was determined through simultaneous saccharification and fermentation (SSF) process using carboxymethyl cellulose and alkali-pretreated bagasse as substrates. 

## 2. Methods

### 2.1. Bacterial Strains, Plasmids, and Growth Conditions

*Z. mobilis* subsp*. mobilis* MTCC 92 [5] and* E. coli* DH5*α* were obtained from the Microbial Type Culture Collection (MTCC), Chandigarh, India.* E. coli* harboring pUC18 *amp* ^R^, cloning vector,* lac* promoter, (2.7 kb) was obtained from Fermentas (USA)*. E. cloacae *JV and* P. mirabilis* JV were isolated and characterized in our laboratory from the gut of termite (*Heterotermes indicola*) and silk worm (*Bombyx mori*), respectively. The 16s rDNA sequence of the organisms was submitted in National Center for Biotechnology Information (NCBI),* E. cloacae* JV (FJ 799063) and* P. mirabilis* JV (HQ231796).* E. chrysanthemi* was obtained from the National Institute of Agrobiological Sciences, Japan.* Z. mobilis* subsp*. mobilis* was grown on yeast extract medium supplemented with 20% glucose, 0.5% yeast extract, 0.1% ammonium sulphate, 0.1% potassium dihydrogen ortho-phosphate, and 0.05% magnesium chloride, pH 7, at 30°C with agitation at 100 rpm.* E. coli* harbouring pUC18 was grown on Luria agar with ampicillin (100 *μ*g/mL) under static condition at 37°C and* E. chrysanthemi, E. cloacae* JV,* P. mirabilis* JV, and* E. coli* DH5*α* were also cultured on Luria agar. 

### 2.2. Cloning of* pdc* and* adh* II Genes

Chromosomal DNA was isolated from* Z. mobilis* MTCC 92 as described by Sambrook and Russel [13] and the pUC18 plasmid DNA was isolated by alkaline lysis method [14]. Cloning of* pdc* gene was carried out by restricting both the total genomic DNA (20 *μ*g) and plasmid DNA (7 *μ*g) with 10 units of* Eco*RI, 10 units of* Bam*HI (Fermentas, USA), 2.5 *μ*L of restriction buffer, and 9.5 *μ*L of sterile distilled water to a total volume of 25 *μ*L. The* adh* II gene cloning was done as described above using* Bam*HI and* Hind*III enzymes. The reaction mixtures were incubated at 37°C for 2 h. The restriction reaction was stopped by heating the reaction mixture at 65°C for 20 min. The restricted and purified DNA samples were ligated by mixing 8 *μ*g of digested genomic DNA, 1 *μ*g of digested pUC18 plasmid DNA, 4 *μ*L of T_4_ DNA ligase buffer, and 5 units of T_4_ DNA ligase enzyme (Fermentas, USA) and incubated at 16°C for 16 h [13]. 

The ligated mix was transformed into competent* E. coli* DH5*α* cells by CaCl_2_ method. The transformants were plated on Luria agar supplemented with ampicillin (50 mg/mL), IPTG (40 mg/mL), and X-gal (20 mg/mL), the white-coloured recombinant clones were selected. The* pdc* clones expressing pyruvate decarboxylase enzyme were further screened by plating the white colonies on Luria agar supplemented with 1% Schiff reagent, 50 mM sodium pyruvate and ampicillin (50 mg/mL) whereas the* adh* clones were screened on Luria agar supplemented with 1% Schiff reagent, 5% ethanol, and ampicillin (50 mg/mL) [15]. The clones showing intensive red colour on aldehyde indicator plates were selected as positive clones. 

### 2.3. Cell Extracts Preparation

The* pdc* and* adh* positive clones were grown in 100 mL Luria broth supplemented with ampicillin (100 mg/mL) for 18 h at 37°C. After incubation, cells were harvested by centrifugation (10,000 rpm, 5 min, 4°C) and the cells were washed with 10 mM Tris hydrochloride buffer (pH 7.0) containing 1 mM EDTA and resuspended in 10 mL of the same buffer. Lysozyme was added at a final concentration of 1 mg/mL and the mixture was incubated for 30 min at room temperature. Cells were lysed by sonication using a Bandelin sonicator (UW 2200) for three cycles at 40 W with 45 sec intervals. Cell extracts were collected by centrifugation (15,000 rpm, 30 min, 4°C) and the supernatant was used as source of enzyme [16]. 

## 3. Expression of* Z. mobilis* Genes in* E. coli*

### 3.1. PDC Activity

PDC activity was measured in triplicate by monitoring the pyruvic acid-dependent oxidation of NADH with ADH as a coupling enzyme. The reaction mixture consisted of 2.7 mL of 200 mM citrate buffer, 100 *μ*L of 1 M sodium pyruvate, 50 *μ*L of 6.4 mM *β*-NADH, and 100 *μ*L of cell extract. The reaction mixture was mixed and the assay was carried out at 25°C. The enzyme activity was determined by measuring the conversion of NADH to NAD^+^ at 340 nm using varian spectrophotometer. The decrease in absorbance value was recorded. The rate that is rA 340 nm/mL was obtained using the maximum linear rate for both the test and the blank [17]. One unit of activity is defined as the amount of activity required for the conversion of 1 *μ*mol of NADH to NAD^+^ per min. 

### 3.2. ADH Activity

Assay of ADH was measured in triplicate by monitoring the ethanol-dependent reduction of NAD, in which the conversion of NAD to NADH was determinedspectrophotometrically. The reaction mixture containing 0.1 mL of 15 mM NADP, 2.4 mL of 100 mM Tris- HCl, 0.3 mL of propane 2-ol (100%), and 0.2 mL of cell extract and incubated at 40°C for 5 min. The alcohol-dependent reduction of NAD^+^ using propane-2-ol was measured at 340 nm [18]. One unit of ADH activity is defined as the amount that reduces 1 *μ*mol of NAD^+^/min. 

Both enzyme activities were calculated using the following formula:

(1) Unit/mL extract=(ΔA340 nm/min test  −  ΔA340 nm/min blank)  (6.22)  (enzyme volume)×(reaction volume)×  DF.

6.22 is the millimolar extinction coefficient of *β*-NADH at 340 nm and DF is the dilution factor. 

### 3.3. DNA Sequencing

The clones which showed higher PDC and ADH activity were selected for sequencing. The cycle sequencing reaction was performed using BigDye Terminator V3.1 Cycle Sequencing Kit containing AmpliTaq DNA polymerase (from Applied Biosystems, PN: 4337457). The sequencing reaction mix was prepared by adding 1 *μ*L of BigDye v3.1, 2 *μ*L of 5x sequencing buffer, and 1 *μ*L of 50% Dimethyl sulfoxide (DMSO). Four microlitres of sequencing reaction mixture, 4 Pico moles of primer (2 *μ*L), and sufficient amount of plasmid DNA were added. The constituted reaction was denatured at 95°C for 5 min. Cycling began with denaturing at 95°C for 30 sec, annealing at 52°C for 30 sec, and extension for 4 min at 60°C and cycle repeated for 30 cycles in a MWG thermocycler. The reaction content was then purified on sephadex plate (Edge Biosystems) by centrifugation to remove unbound labeled, and unlabeled nucleotides and salts. The purified reaction product was loaded on to the 96 capillary ABI 3700 DNA analyzer and electrophoresis was carried out for 4 h. The nucleotide sequences of both* pdc* and* adh* II genes were analysed, confirmed, and submitted to the National Center for Biotechnology Information (NCBI). 

### 3.4. Cloning of* pdc* and* adh* II Genes

Both* pdc* and* adh* II genes were subcloned together by digesting pUC18-*adh* with *Bam*HI and* Hind*III enzymes and eluted from the gel to ligate with* Bam*HI- and* Hind*III-digested pUC18-*pdc* clone in such a way that the* adh* II fragment was at the downstream of *pdc* gene. The ligated mix was transformed into competent* E. coli* DH5*α* cells by calcium chloride method. The transformants having both ADH and PDC activity were screened further on selective aldehyde indicator plates, enzyme assays, and by restriction analysis [13]. 

### 3.5. Ethanol Tolerance Assay

Single colony of each cellulolytic bacteria such as* E*.* chrysanthemi, E. cloacae* JV, and* P. mirabilis* JV were inoculated separately in 5 mL of Luria broth and incubated at 37°C in a shaker at 200 rpm. Five hundred microlitres of the overnight cultures were subcultured to 50 mL Luria broth (supplemented with 0, 2, 4, 6, and 10% ethanol) in closed culture tubes to prevent ethanol volatilization and were incubated at 37°C on a rotary shaker with an aeration speed of 200 rpm and the density of bacterial culture was measured at 600 nm [19]. 

### 3.6. Transformation of Cellulolytic Bacteria

The pUC18-*pdc*-*adh* II plasmid was purified from* E. coli* and transformed into cellulolytic bacteria such as* E*.* chrysanthemi, E. cloacae *JV, and* P. mirabilis* JV by electroporation (single pulse at 6.25 KV using 25 mF capacitor at a resistance of 200 Ohm in cooled 0.2 cm cuvette which contained 50 ng of plasmid DNA, 40 *μ*L of* P. mirabilis* JV*, E. cloacae* JV, and* E*.* chrysanthemi* competent cells in separate cuvettes using Biorad electroporator). After electroporation, cells were incubated for one hour in SOC medium and then plated on selective agar supplemented with ampicillin (100 *μ*g/mL) [20]. Those clones that developed intensive red color on aldehyde indicator plates were selected. The clones showing higher enzymatic activity were further confirmed by PDC/ADH assay. 

### 3.7. Analysis of Plasmid Profile and Restriction Mapping

The transformants of cellulolytic bacteria were confirmed by analyzing their plasmid profile and by restriction analysis. The transformation of plasmid pUC18-*pdc*-*adh* into the cellulolytic bacteria was confirmed through horizontal slot lysis electrophoresis as described by Vennison [21]. The transformed colonies on agar plates were resuspended in protoplasting buffer (15 *μ*L) to a density of 10^5^ cell/mL. Bacterial cells were mixed thoroughly by vigorous vortexing. The mixtures were incubated at 37°C for 15 min for the formation of protoplasts. Agarose gel (0.7%) was prepared with 1X Tris-Boric acid-EDTA buffer with 0.05% SDS. The gel slots were preloaded with 20 *μ*L of lysis buffer and allowed to stand for 20 min. Then 10 *μ*L of protoplast suspension was loaded into each slot and the electrophoresis was carried out initially with 50 volts and then to 100 volts till the completion of the run. After the completion of electrophoresis, the gel was stained with 0.05 *μ*g/mL of ethidium bromide. The size of plasmids such as pUC18-*pdc*, pUC18-*adh,* and pUC18-*pdc*-*adh* was determined by linearizing the plasmids with* Bam*HI enzyme and electrophoresed on 0.7% of agarose gel along with the DNA molecular weight marker. The DNA bands were visualized under UV transilluminator and photographed using Alpha gel documentation system (USA). 

### 3.8. Cellulosic Ethanol Production

Ethanol fermentation experiments were carried out independently with 0.6% carboxyl methyl cellulose and 1 g of 4% NaOH-treated bagasse [5] in the luria broth supplemented with 0.1% ammonium sulfate, 0.1% potassium dihydrogen orthophosphate, and 0.05% magnesium sulphate at 37°C and pH 7.0 with an agitation speed of 150 rpm agitation as described by Jeffers [22]. The fermentation was performed in a round bottom flask connected with an *U*-tube. The outlet was fitted with a test tube containing Ca(OH)_2_ to maintain anaerobic conditions and pH of the fermentation medium [23]. After 48 h, the ethanol was distilled at 78.5°C and ethanol concentrations in the distillate were determined by potassium dichromate method [24]. 

## 4. Results 

### 4.1. Cloning of* pdc* and* adh* II Genes and Their Expression in* E. coli*

**Figure 1:**
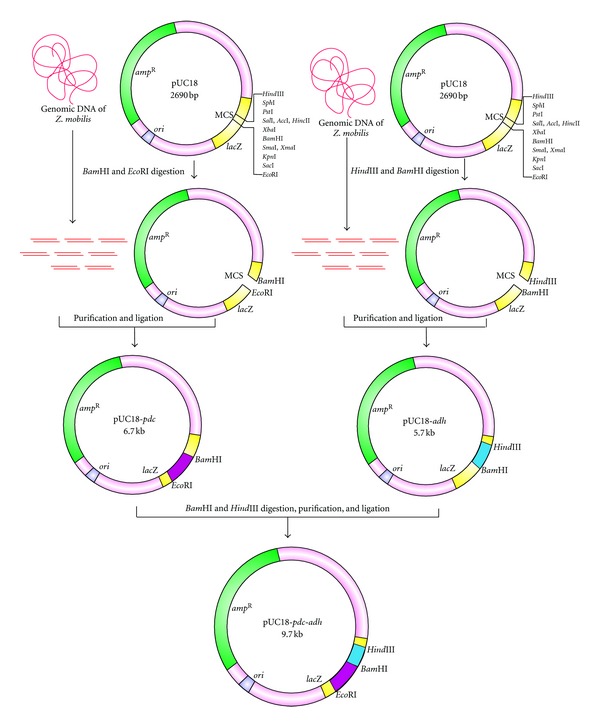
Cloning of* pdc* and *adh* II genes in pUC18 plasmid.

**Figure 2:**
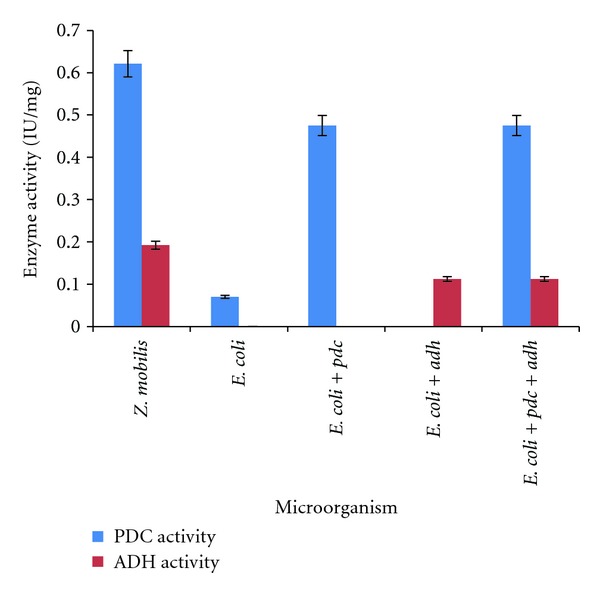
The intracellular PDC and ADH activities of the recombinant bacteria.

**Figure 3:**
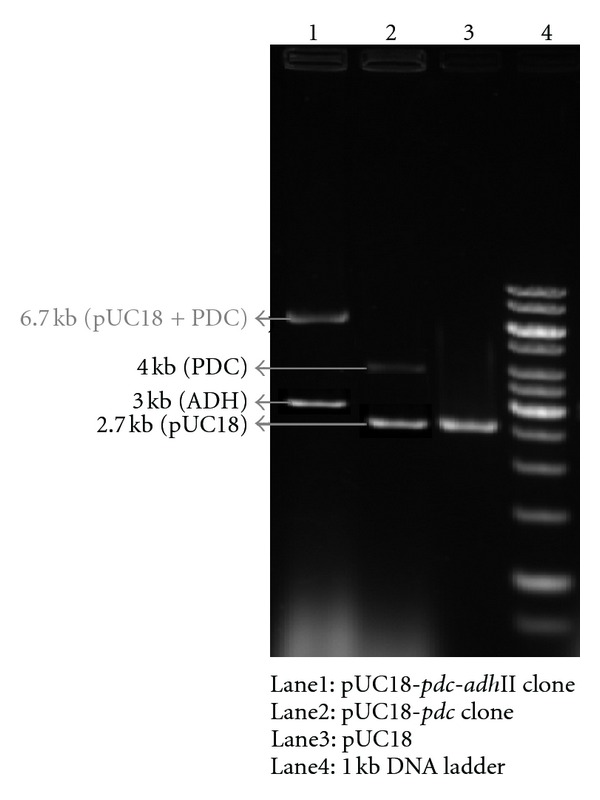
Restriction analysis of *pdc* and *adh* II clones.

*Z. mobilis* genes encoding PDC and ADH II enzymes were expressed in* E. coli* using a vector pUC18 (Figure 1) and the transformants expressing the gene were screened by red spots on aldehyde indicator plates supplemented with ampicillin (100 mg/mL). The PDC and ADH activity was further confirmed by direct spectrophoto metric assay of cell lysate (Figure 2). A PDC clone showed a higher activity of 0.6582 (U/mL) was named as pUC18-*pdc,* whereas an ADH clone showed an activity of 0.117 (U/mL) was named as pUC18-*adh* II. The experiment was repeated for six times and the enzyme activity data were statistically analyzed by Student's *t*-test by comparing the enzyme activity of the clone with control* E. coli* strain. The statistical analysis predicted that the calculated value for both enzyme activities was greater than the tabulated value (10.1 > 2.36) at *P* < 0.05. These analyses showed that there was a significant difference between the enzyme activities of the clones and the control* E. coli* strain. The nucleotide sequences of both* pdc* and* adh* II genes cloned from* Z. mobilis* were deposited in NCBI (the accession number for* pdc* gene is HM235920 and for *adh* gene is HM235921). The* pdc* gene sequence contained an open-reading frame of 1707 bp and the* adh* II gene contained an open-reading frame of 1152 bp. Both* pdc* and* adh* II gene sequences showed a maximum of 99% similarity when compared to the sequence of* pdc* and *adh* II gene of* Z. mobilis* already available in NCBI (AB359062.1 and AB359063.1). These clones of* pdc* and* adh* genes were found to contain insert DNA of 3 and 4 kb, respectively (Figure 3). 

### 4.2. Construction of pUC18-*pdc*-*adh* for Ethanol Production

The pUC18-*adh* II and pUC18-*pdc* were digested with the restriction enzymes* Bam*HI and* Hind*III, ligated and transformed into* E. coli*. Clones expressing both* pdc* and* adh* II genes grew poorly on Luria agar plates but grew at higher densities than the individual* pdc *and* adh* II clone on agar plates supplemented with 2% glucose. The colony size and opacity had proven as useful markers for the identification of recombinants which harboured both alcohol dehydrogenase and pyruvate decarboxylase genes. Positive colonies appeared intensely red, whereas negative colonies ranging from white to medium shades of red on the aldehyde indicator plate. Among the 20 positive colonies, five intensively red colonies were selected and their intracellular enzyme activities were determined. The efficient clone with higher enzyme activity was designated as pUC18-*pdc*-*adh* and selected for further studies. 

### 4.3. Ethanol Tolerance Assay

**Figure 4:**
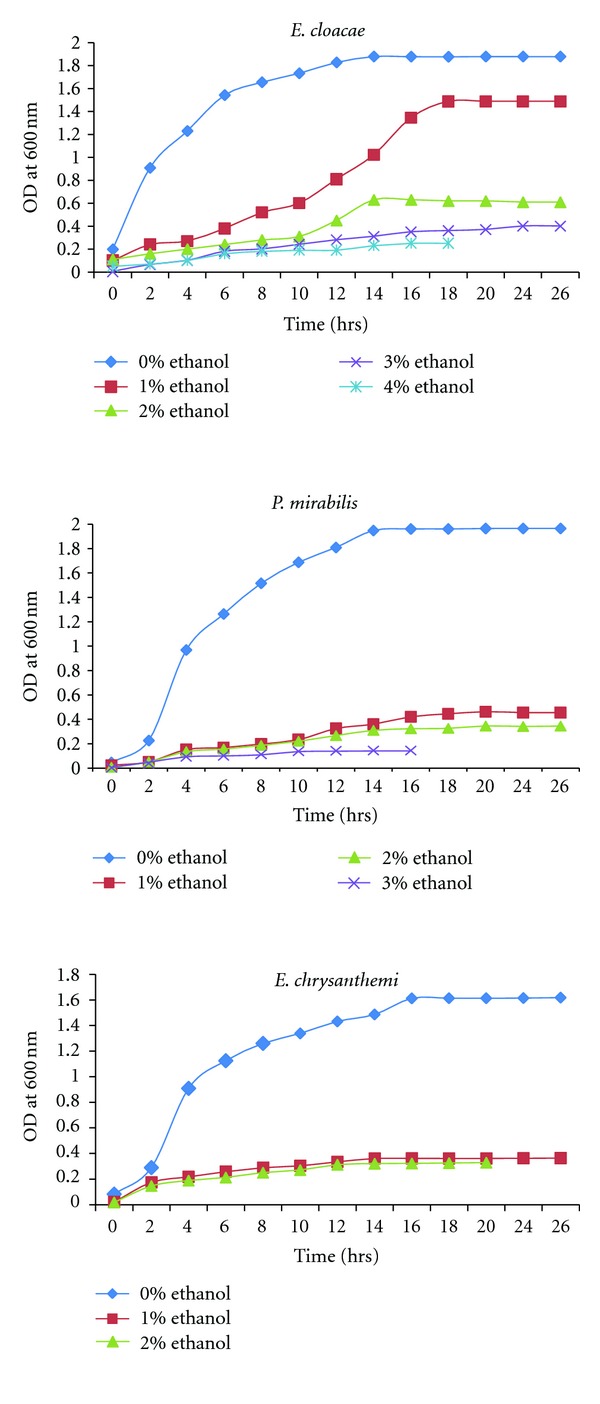
Ethanol tolerance assay of cellulolytic bacteria.

The ethanol tolerance assay of cellulolytic bacteria was carried out by culturing the bacterial strains in the luria broth supplemented with ethanol at different concentration (0–10%) and the culture densities were measured at 600 nm (Figure 4). The turbidity of* E. cloacae* was clearly visible till 4%, whereas the turbidity of the other strains was visible only till 2% of ethanol. The optical density of these cellulolytic bacteria in different concentration of ethanol medium revealed that the* E. cloacae* growth rate was decreased slowly till 4%, but the growth rates of other strains were rapidly decreased at 1-2%. 

### 4.4. Transformation of pUC18-*pdc*-*adh* into Cellulolytic Bacteria

The pUC18-*pdc*-*adh* clone was transformed into* E*.* chrysanthemi, E. cloacae* JV, and *P. mirabilis* JV through electroporation. The transformants were selected on the aldehyde indicator plates supplemented with ampicillin. On agar medium, the recombinant ethanologenic clones were readily apparent as large, raised colonies. The efficient strains that are able to convert glucose to ethanol were recognized by the production of red spots on aldehyde indicator plates. Efficient clones from each cellulolytic strain with efficient enzyme activity were selected for cellulosic ethanol production. Plasmid DNA profile from all the three recombinant cellulolytic bacteria was examined through slot lysis electrophoresis which was found identical to that of pUC18-*pdc*-*adh*. 

### 4.5. Fermentation of Cellulose to Ethanol

**Figure 5:**
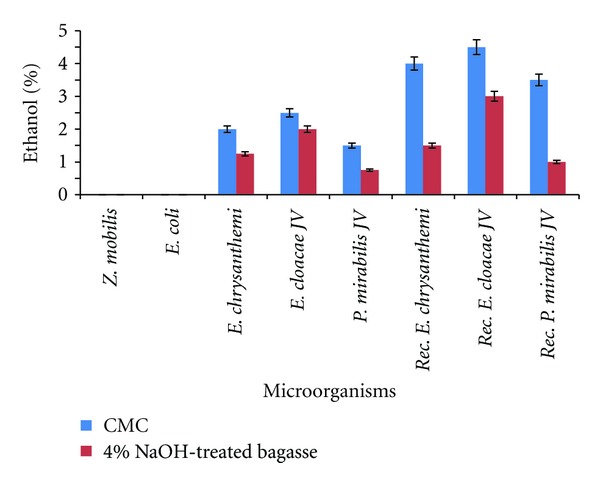
Cellulosic ethanol production in wild type and recombinant cellulolytic bacteria using CMC and 4% NaOH-treated bagasse.

The optimum temperature and pH for the ethanol production was 37°C and 7.0. The fermentation was carried out under anaerobic conditions for 48 h with agitation of 150 rpm. The cellulosic ethanol production capability of recombinant cellulolytic bacteria harbouring both *pdc* and* adh* II genes was studied with carboxymethyl cellulose and pretreated bagasse as substrates (Figure 5). The recombinant strains produced ethanol more rapidly and efficiently when compared to their respective parental strains. Recombinant* E. chrysanthemi* could produce ethanol from CMC and pretreated bagasse slightly higher than the wild type. The ethanol production by recombinant* P. mirabilis* from 4% NaOH-treated bagasse did not show any significant increase when compared to the wild type. The recombinant* E. cloacae* JV had shown twofold increase in ethanol production than the wild type. Among the recombinants *E. cloacae* JV harboring pUC18-*pdc*-*adh,* plasmid construct was identified as the best strain for ethanol production, with a maximum of 4.5% and 3.5% of ethanol with carboxyl methyl cellulose and 4% NaOH treated bagasse, respectively. Experiments were performed six times and statistical analyses of the data were performed using the Student's *t*-test. The statistical analyses showed a significant difference in the cellulosic ethanol production between the wild type* E. cloacae* and recombinant* E. cloacae* at *P* < 0.05. 

## 5. Discussion

Cellulosic ethanol production from lignocellulosic biomass is a globally developing technology. One of the major issues for cellulosic ethanol production is enzyme hydrolysis by the naturally available strains to convert cellulose to glucose. Developing a single strain for efficient cellulosic ethanol production is the technical challenge. The present work has taken up the challenge by improving the ethanol fermenting capabilities of cellulolytic bacteria through cloning of* pdc* and* adh* genes from* Z. mobilis*. 

There are several reports on the construction of an artificial,* pet* operon, for the production of ethanol by combining both* pdc* and* adh*II genes. The first successfully constructed recombinant organism was* E. coli* KO11 which had the ability to ferment a wide spectrum of sugars but the ethanol yield was 4.3% from glucose as a substrate [9], but the cells could tolerate only 2% ethanol [25]. Other Gram-negative bacteria such as* Klebsiella oxytoca* and* E. chrysanthemi* were also transformed with* pet* operon but these strains have lower ethanol yield than* E. coli* KO11 [26]. The* K*.* oxytoca* was further improved to enhance the ethanol yield by overcoming its limitations, but the yield was increased to 40 g/L using raw sugarcane but the process took 13 days time for the overall production [27]. The expression of this* pet* operon in other Gram-positive microorganisms also had shown very less ethanol yield [28]. The engineered cellulolytic bacterium,* Clostridium cellulolyticum* with* pdc* and *adh* II of* Z. mobilis* showed 150% increase in cellulose consumption and the concentrations of acetate and ethanol increased by 93 and 53%, respectively, [12] but the drawback of this strain was its slower growth rate than the wild type. The major limitations in all those recombinants for cellulosic ethanol production were intracellular cellulase enzyme activity, low ethanol yield, and their inability to tolerate higher percentage of ethanol. The present work focused on the cellulose hydrolysis by the microbial enzymes and fermentation of the hydrolyzed products into ethanol. The* pdc* and* adh* clones of cellulolytic bacteria were screened on aldehyde indicator plates by adding acetaldehyde as a substrate for* adh *and ethanol for* pdc*. The* pdc* clones had showed a PDC activity of 0.6582 U/mL and the* adh* II clones showed an ADH activity of 0.117 U/mL. 

The cellulolytic capability of insect gut-inhabiting bacteria was higher because they naturally involved in the digestion of lignocellulosic substrates which is the diet of insects. The selected cellulolytic bacteria used in the present study were isolated from various phytophagous insects so that they were efficient in cellulolytic activity. The microorganisms such as,* E*.* chrysanthemi*,* E. cloacae,* and* P. mirabilis *were already known for their cellulolytic activity [20, 29]. Cloning of cellulase gene into ethanologenic bacteria had also already been reported [5]. The ethanol tolerance capability was studied to detect the effect of ethanol on the growth of microorganisms.* E. cloacae* exhibited the growth up to 4% (v/v) supplemented ethanol and the other strains such as* E*.* chrysanthemi* and* P. mirabilis* were found to grow less rapidly in all ethanol concentrations, whereas there was no growth observed in 4%. Addition of zinc in fermentation medium was found to increase the tolerance towards ethanol was reported [30] and over expression of genes involved in tryptophan biosynthesis and/or supplementation of tryptophan in the fermentation medium could also reportedly improve ethanol tolerance in yeast [31]. 

The ethanol production capability of the recombinant microorganisms was studied using fermentation medium under anaerobic conditions with carboxymethyl cellulose and 4% NaOH pretreated bagasse as substrates. Production of high levels of PDC and ADH enzymes metabolically diverts pyruvate to ethanol as the primary product of fermentation. Expression of these enzymes for ethanol production was simultaneously increased, as evidenced by the increase in PDC activity, stronger reaction on aldehyde indicator plates (ADH II), decreased acetate, and more efficient ethanol production. As the sugar released by the cellulase action was subsequently utilized for ethanol production, as the feedback inhibition was minimum. The ethanol production of recombinant* E*.* chrysanthemi* and * P. mirabilis* using CMC was 3.5% and 3%, respectively, whereas using 4% NaOH-treated bagasse ethanol production was less than 2%. The ethanol production of recombinant * E*.* chrysanthemi* and* P. mirabilis* JV from 4% NaOH-pretreated bagasse has no significant difference from that of the wild type. The ethanol production of recombinant* E. cloacae* from CMC and 4% NaOH treated bagasse were 4.5 and 3.5%, which are higher than the other cellulolytic bacterial strains studied. This might be due to its superior ethanol tolerance, wide substrate utilization, and higher cellulolytic activity. The recombinant* E. cloacae* can be improved further by studying sugar catabolism and nutrient requirements to increase its ethanol production. 

## 6. Conclusion

Three recombinant cellulolytic bacterial strains such as* E. cloacae* JV,* E. chrysanthemi, *and* P. mirabilis* harbouring both* pdc* and* adh* II genes from* Z*.* mobilis* have showed an increase in cellulosic ethanol production capabilities when compared to their respective wild-type strains. Recombinant* E. cloacae* JV harboring both* pdc* and* adh* II genes produced 4.5 and 3.5% of ethanol when CMC and 4% NaOH-treated bagasse were used as substrates, but recombinant* E. chrysanthemi* produced 4 and 1.5% and recombinant* P. mirabilis* produced 3.5 and 1% ethanol using the same substrates, respectively. The cellulosic ethanol production could be increased by over expressing the genes and optimizing the fermentation conditions for altering cellular metabolism for higher ethanol tolerance. 

## References

[1] Zaldivar J, Nielsen J, Olsson L (2001). Fuel ethanol production from lignocellulose: a challenge for metabolic engineering and process integration. *Applied Microbiology and Biotechnology*.

[2] Ohta K, Beall DS, Mejia JP, Shanmugam KT, Ingram LO (1991). Metabolic engineering of *Klebsiella oxytoca* M5A1 for ethanol production from xylose and glucose. *Applied and Environmental Microbiology*.

[3] Wood BE, Yomano LP, York SW, Ingram LO (2005). Development of industrial-medium-required elimination of the 2,3-butanediol fermentation pathway to maintain ethanol yield in an ethanologenic strain of *Klebsiella oxytoca*. *Biotechnology Progress*.

[4] Yanase H, Nozaki K, Okamoto K (2005). Ethanol production from cellulosic materials by genetically engineered *Zymomonas mobilis*. *Biotechnology Letters*.

[5] Vasan PT, Piriya PS, Prabhu DIG, Vennison SJ (2011). Cellulosic ethanol production by *Zymomonas mobilis* harboring an endoglucanase gene from *Enterobacter cloacae*. *Bioresource Technology*.

[6] Beall DS, Ingram LO (1993). Genetic engineering of soft-rot bacteria for ethanol production from lignocellulose. *Journal of Industrial Microbiology*.

[7] Wills C, Kratofil P, Londo D, Martin T (1981). Characterization of the two alcohol dehydrogenases of *Zymomonas mobilis*. *Archives of Biochemistry and Biophysics*.

[8] Neale AD, Scopes RK, Kelly JM, Wettenhall RE (1986). The two alcohol dehydrogenases of *Zymomonas mobilis*: purification by differential dye ligand chromatography, molecular characterisation and physiological roles. *European Journal of Biochemistry *.

[9] Ingram LO, Conway T, Clark DP, Sewell GW, Preston JF (1987). Genetic engineering of ethanol production in *Escherichia coli*. *Applied and Environmental Microbiology*.

[10] Lee AT, Malgorzata AG, Lorraine PY, Ingram LO, Julie AM (2005). Construction and expression of an ethanol production operon in Gram-positive bacteria. *Microbiology*.

[11] Tolan JS, Finn RK (1987). Fermentation of D-Xylose and L-arabinose to ethanol by *Erwinia chrysanthemi*. *Applied and Environmental Microbiology*.

[12] Guedon E, Desvaux M, Petitdemange H (2002). Improvement of cellulolytic properties of *Clostridium cellulolyticum* by metabolic engineering. *Applied and Environmental Microbiology*.

[13] Sambrook J, Russel DW (2003). *Molecular Cloning: A Laboratory Manual*.

[14] Bimboim HC, Doly J (1979). A rapid alkaline extraction procedure for screening recombinant plasmid DNA. *Nucleic Acids Research*.

[15] Lillie RD (1977). *H. J. Conn's Biological Stains*.

[16] Gunasekaran P, Karunakaran T, Cami B, Mukundan AG, Preziosi L, Baratti J (1990). Cloning and sequencing of the sacA gene: characterization of a sucrase from *Zymomonas mobilis*. *Journal of Bacteriology*.

[17] Gounaris AD, Turkenkopf I, Buckwald S, Young A (1971). Pyruvate decarboxylase. I. Protein dissociation into subunits under conditions in which thiamine pyrophosphate is released. *The Journal of Biological Chemistry*.

[18] Fibla J, Atrian S, Duarte RG (1993). Evidence of serine-protease activity closely associated with Drosophila alcohol dehydrogenase. *European Journal of Biochemistry*.

[19] Zhou Y, Ye WX, Zhou Y, Zhu CG, Sun M, Yu ZN (2006). Ethanol tolerance, yield of melanin, swarming motility and growth are correlated with the expression levels of aiiA gene in Bacillus thuringiensis. *Enzyme and Microbial Technology*.

[20] Fernandes BL, Da Costa SOP (1996). High efficiency of transformation of *Proteus mirabilis* with a pUC19 derivative vector directs the expression and secretion of the Bacillus subtilis *α*-amylase gene. *Journal of Microbiological Methods*.

[21] Vennison SJ (2009). *Laboratory Manual for Genetic Engineering*.

[22] Jeffers J (2000). *Preparation of Ethanol by Fermentation*.

[23] Fogel S, Lancione RL, Sewall AE (1982). Enhanced biodegradation of methoxychlor in soil under sequential environmental conditions. *Applied and Environmental Microbiology*.

[24] Kiransree N, Sridhar M, Rao LV (2000). Characterisation of thermotolerant, ethanol tolerant fermentative *Saccharomyces cerevisiae* for ethanol production. *Bioprocess Engineering*.

[25] Hilaly AK, Karim MN, Linden JC (1994). Use of an Extended Kalman Filter and development of an automated system for xylose fermentation by a recombinant *Escherichia coli*. *Journal of Industrial Microbiology*.

[26] Lawford HG, Rousseau JD (1996). Factors contributing to the loss of ethanologenicity of *Escherichia coli* B recombinants pLOI297 and KO11. *Applied Biochemistry and Biotechnology—Part A*.

[27] Doran JB, Cripe J, Sutton M, Foster B (2000). Fermentations of pectin-rich biomass with recombinant bacteria to produce fuel ethanol. *Applied Biochemistry and Biotechnology—Part A*.

[28] Senthilkumar V, Gunasekaran P (2005). Bioethanol production from cellulosic substrates: engineered bacteria and process integration challenges. *Journal of Scientific and Industrial Research*.

[29] Anand AP, Vennison SJ, Sankar SG (2009). Digestion of cellulose, pectin, xylan and starch by the symbiotic gut bacteria in the intestine of*Bombyx mori*. *Insect Science*.

[30] Zhao XQ, Xue C, Ge XM, Yuan WJ, Wang JY, Bai FW (2009). Impact of zinc supplementation on the improvement of ethanol tolerance and yield of self-flocculating yeast in continuous ethanol fermentation. *Journal of Biotechnology*.

[31] Yazawa H, Iwahashi H, Uemura H (2007). Disruption of URA7 and GAL6 improves the ethanol tolerance and fermentation capacity of *Saccharomyces cerevisiae*. *Yeast*.

